# Identification of 5-Iodotubercidin as a Genotoxic Drug with Anti-Cancer Potential

**DOI:** 10.1371/journal.pone.0062527

**Published:** 2013-05-07

**Authors:** Xin Zhang, Deyong Jia, Huijuan Liu, Na Zhu, Wei Zhang, Jun Feng, Jun Yin, Bin Hao, Daxiang Cui, Yuezhen Deng, Dong Xie, Lin He, Baojie Li

**Affiliations:** 1 Bio-X Institutes, Key Laboratory for the Genetics of Developmental and Neuropsychiatric Disorders, Ministry of Education, Shanghai Jiao Tong University, Shanghai, China; 2 Instrumental Analysis Center, Shanghai Jiao Tong University, Shanghai, China; 3 School of Life Sciences and Biotechnology, Shanghai Jiao Tong University, Shanghai, China; 4 School of Pharmacy, Shanghai Jiao Tong University, Shanghai, China; 5 Research Institute of Micro/Nano Science and Technology, Shanghai Jiao Tong University, Shanghai, China; 6 Institute for Nutritional Sciences, Shanghai Institutes for Biological Sciences, Chinese Academy of Sciences, Shanghai, China; Rush University Medical Center, United States of America

## Abstract

Tumor suppressor p53, which is activated by various stress and oncogene activation, is a target for anti-cancer drug development. In this study, by screening panels of protein kinase inhibitors and protein phosphatase inhibitors, we identified 5-Iodotubercidin as a strong p53 activator. 5-Iodotubercidin is purine derivative and is used as an inhibitor for various kinases including adenosine kinase. We found that 5-Iodotubercidin could cause DNA damage, verified by induction of DNA breaks and nuclear foci positive for γH2AX and TopBP1, activation of Atm and Chk2, and S15 phosphorylation and up-regulation of p53. As such, 5-Iodotubercidin induces G2 cell cycle arrest in a p53-dependent manner. Itu also induces cell death in p53-dependent and -independent manners. DNA breaks were likely generated by incorporation of 5-Iodotubercidin metabolite into DNA. Moreover, 5-Iodotubercidin showed anti-tumor activity as it could reduce the tumor size in carcinoma xenograft mouse models in p53-dependent and -independent manners. These findings reveal 5-Iodotubercidin as a novel genotoxic drug that has chemotherapeutic potential.

## Introduction

Cancer is one of the leading causes of mortality worldwide. According to the recently released data, there were 12.7 million new cancer cases and 7.6 million cancer patients died in 2008, with breast cancer being most common in females and lung cancer most common in males [1]. Cancer treatment includes surgical procedures to remove malignant tissue and the use of radiotherapy and chemotherapy to kill malignant cells and/or curtail tumor growth. Chemotherapeutic drugs are classified into alkylating agents, anti-metabolites, topoisomerase inhibitors, plant alkaloids and terpenoids, anti-tumor antibiotics, and others [2]. Up to now, there is a lack of fail-proof treatment for most kinds of cancer [3].

Anti-metabolites are derivatives of nucleosides, including pyrimidine analogs. e.g., gemcitabine and purine analogs, e.g., fludarabine [4]. They can be incorporated into the DNA, leading to termination of nascent DNA strand extension. Some nucleoside analogs can also inhibit the enzymes involved in DNA synthesis or enzymes that maintain deoxynucleotides homeostasis, further interfering with DNA synthesis. As such, many of these nucleoside analogs cause DNA damage including double and single stranded DNA breaks to trigger the internal defense mechanisms to kill rapid dividing cells or cells undergoing active DNA repair, or cause cell cycle arrest at the S phase [4]. This is a major mechanism by which most anti-metabolites execute their anti-cancer activity.

In cells, DNA damage or genotoxic drug activates a family of PI3K like kinases (PIKKs) including Atm, Atr, and DNA-PKcs at the DNA breaks or stalled replication forks, where they phosphorylate various proteins including γH2AX, TopBP1, Chk1/2 and p53. The integrated activation of these effector proteins induces cell cycle arrest, cell senescence or apoptosis [5], [6]. As a result, cells with unstable genome are eliminated, which prevents tumor formation [7]–[11]. The Atm-p53 pathway is a major tumor suppressing pathway and p53 is mutated in more than 50% of the human primary tumors [12]–[14].

p53 activation promotes cell cycle arrest, senescence and apoptosis. Activation or up-regulation of p53 in tumors has been shown to inhibit tumor growth, establishing p53 as a target for anti-cancer drug development [13], [15]. Several small molecule compounds have been developed that specifically target Mdm2-p53 interaction, stabilize p53, and inhibit tumor growth. In addition, p53 can be delivered to tumors with viruses and this has been shown to have significant therapeutic effects [16]. To search for new potential anti-tumor drugs, we screened small molecule inhibitors of a panel of protein kinases and a panel of protein phosphatases for compounds that could activate p53 [17]. Here we report the identification of 5-Iodotubercidin (Itu) as a p53 activator. Itu is being used as a general kinase inhibitor, especially adenosine kinase (ADK) due to its affinity for the ATP-binding sites of these enzymes and has been shown to affect cell proliferation and survival. ADK catalyzes the transfer of the γ-phosphate group of ATP to adenosine, thereby down-regulating the cellular levels of adenine nucleotides [18]–[20]. Itu has a structure similar to adenosine and was shown here to generate DNA damage, activate the Atm-p53 pathway, and induce cell cycle arrest at G2 phase in p53-dependent manners. Itu also promotes cell death in p53-dependent and -independent manners. More importantly, Itu was found to have anti-tumor activity, also in a p53-dependent and -independent manners. These results suggest that Itu is a potential chemotherapeutic drug with properties distinct from most other anti-metabolites. Since Itu executes its anti-tumor activity mainly via generating DNA damage, Itu may also cause genome instability in normal cells and potentially lead to the development of cancer. Thus, caution should be exercised when using Itu for potential non-cancer related therapeutic purpose.

## Materials and Methods

### Ethics Statement

Animal experimentation in this study, including BALB/cASlac nude mice, normal C57B/6 mice, Atm+/− mice, was carried out in accordance with recommendations in the National Research Council Guide for Care and Use of Laboratory Animals, with the protocols approved by the Institutional Animal Care and Use Committee of Shanghai, China [SYXK (SH) 2011–0112]. All efforts were made to minimize suffering of mice.

### Cell Culture

The primary mouse embryo fibroblast (MEF) cells (from C57B/6 mice) and Atm−/− MEFs (from 129 mice) were generated in the laboratory as described previously [11]. The human colon cancer cell lines HCT116 (p53+/+) and HCT116 (p53−/−) were a gift from B. Vogelstein’s lab [21], [22]. These cells were cultured in DMEM supplemented with 10% heat-inactivated fetal bovine serum (Hyclone) at 37°C in a humidified atmosphere with 5% CO_2_.

### Western Blot Analysis

Cells were lysed in TNEN buffer (50 mM Tris, 150 mM NaCl, 5 mM EDTA, 0.5% NP-40, and 0.1% Triton X-100) supplemented with 1 mM NaF, Na_2_VO_3_, 1 mM PMSF, and 1 µg/ml of aprotonin, leupeptin, and pepstatin A. Protein concentration was determined using a Bio-Rad assay. Proteins were resolved by SDS-PAGE and transferred to polyvinylidene difluoride membranes (Millipore). Antibodies against p-Atm, p-Chk2 (Thr68), Chk2, p-p53 (Ser15), or p53 were obtained from Cell Signaling. Antibodies against Atm (GTX70103) were from Genetex. Antibodies against β-actin were from Santa Cruz Biotechnology.

### RNA Silencing

Small interference RNA (siRNA) targeting human ADK were obtained from GenePharma company and were transfected into HCT116 cells following the manufacturer’s protocol. Down-regulation of ADK was evaluated by realtime PCR 72 hrs after transfection. The sequences of siGENOME ADK SMARTpool were AGGGAGAGAUGACACUAUA; GGAGAGAUGACACUAUAAU; AAAGUUAU GCCUUAUGUUG; and GAGAGAUGACACUAUAAUG. Negative control UUCU CCGAACGUGUCACGUTT; ACGUGACACGUUCGGAGAATT.

### Immunofluorescence Histochemistry

MEFs or HCT116 cells cultured on cover slips were washed with phosphate-buffered saline (PBS) twice and then fixed in 4% paraformaldehyde (PFA), which were permeabilized with 0.1% Triton X in PBS for 30 minutes at room temperature. The primary antibodies were diluted in PBS with 1% BSA (1∶200). The slides were blocked (1% BSA in PBS) for 60 minutes at room temperature, incubated with primary antibodies overnight at 4°C, and followed by secondary antibody incubation for 60 min at RT. The slides were then mounted and observed under confocal microscope.

### RNA Extraction and Realtime PCR

RNAs from cells were extracted using Trizol (Invitrogen). cDNA was synthesized using a Quantscript RT Kit (Tiangen). The mRNA levels of interest were determined by real-time PCR, and normalized to the levels of β-actin. Realtime PCR was carried out using Applied Biosystems 7500 system.

### Cell Cycle Analysis

Cells were seeded on 35-mm dishes and cultured for 24 hrs, reaching 60–70% confluency. The cells were treated with different concentrations of Itu for 24 or 48 hrs, harvested by trypsinization, resuspended in 200 µl PBS, and fixed in 100% ethanol overnight at 4°C. The fixed cells were pelleted by centrifugation, resuspended in 800 µl PBS containing ribonuclease A (100 µg/ml) and incubated for 30 min at 37°C. Then 10 µl propidium iodide (PI, 4 mg/ml PBS) were added to the samples, which were assessed on a FACSCalibur ﬂow cytometer using Cell Quest software (BD Bioscience).

### Cell Survival Assay

To measure cell survival rates after Itu treatment, cells were plated at 1×10^4^ in 96 well plates and cultured for overnight, which were then treated with Itu for 48 hrs. Cell proliferation reagent WST-1 (Roche) was added to each well and was further incubated for 4 hrs at 37°C. The absorbance was measured against a control using microplate reader at 440 nm. The reference wavelength was 630 nm.

### Carcinoma Xenograft Mouse Models

Male BALB/cASlac-nude mice (3 week old) were purchased from Shanghai Slac Laboratory Animal C. LTD. The mice were kept in the SPF mouse facility for 1 week before being inoculated with HCT116 cells. HCT116 (p53+/+ and p53−/−) cells were harvested by trypsinization, counted and resuspended in PBS at the concentration of 5×10^7^/ml. We injected subcutaneously 3×10^6^ cells to BALB/cASlac nude mice’s back, with p53+/+ cells on the left side and p53−/− cells on the right side. After 2 weeks, the mice were divided into several groups and were treated with Itu or solvent (PBS). The mice were weighed and the size of tumor measured. The length (L) and width (W) of the tumor were measured by a digital caliper and expressed as tumor volume (0.5L×W^2^, mm^3^).

### Genomic DNA Analysis by HPLC-MS

MEF cells and HCT116 cells in stationary or log phase were treated with Itu for different periods of time. Genomic DNA was extracted with phenol/chloroform, washed thoroughly with 70% ethanol, and then digested using a previously reported method with modifications [23]. Briefly, aliquots of genomic DNA were incubated with 1 µl DNase I (2 U/µl, NEB), 10 µl Snake Venom Phosphodiesterase (0.26 mU/µl, Sigma Aldrich), and 2 µl Antarctic Phosphatase (5 U/µl, NEB) overnight at 37°C. The complete digestion gave rise to a mixture of mononucleosides, judged by HPLC. The digested DNA samples were analyzed on an HPLC–MS system equipped with an Agilent C18 column (Agilent, San Jose, CA, USA). Solvent A was water containing 0.5% (v/v) formic acid and solvent B was methanol containing 0.25% (v/v) formic acid. An elution profile was used of 2–20% B over 30 min increasing to 98% over another 20 min, then 98% B for 10 min, and finally returning to 2% B over 20 min. The flow rate was set at 20 µl/min and the eluate was monitored at 254 nm. Typically, 5 µl of each sample were injected using the well-plate sampler.

Mass spectrometric analysis of the samples form the HPLC system was carried out directly with an Agilent ESI-TOF mass spectrometer. Mass spectral data were recorded in positive ion mode over the entire duration of the HPLC run. Data were analyzed using Analyst QS (Agilent, San Jose, CA, USA).

### Analysis of the Cellular dNTP Levels

A previously validated LC–MS/MS approach was utilized to determine dNTP concentrations [24]. Standard solutions of dATP and dGTP (dCTP and dTTP were left out due to technical difficulties using this system) at a concentration of 100 mmol/l were utilized to construct a 0, 19.5, 39, 312.5, 625, 1250 and 2500 ng/ml standard calibration curve.

The cells were collected and were resuspended in 500 µl of ice-cold 60% methanol, vortexed, and placed at 95°C for 3 min and sonicated for 30 s in a Branson Sonifier 450 (Branson, Danbury, CT, USA). The extracts were centrifuged at 16000 g for 5 min at 4°C to remove cell debris and precipitated protein and DNA. The resultant supernatants were passed through preequilibrated Amicon Ultra-0.5-ml centrifugal filters at 4°C to remove macromolecules (>3 kDa) following the protocol from the manufacturer (Millipore, Billerica, MA, USA). The filtrate was evaporated under centrifugal vacuum at −70°C and the resultant pellet was resuspended in 25 µl nuclease-free water ready to assay or stored at −80°C until use. Before HPLC analysis, the samples were vacuum-dried again, suspended in 0.5 ml of HPLC H_2_O which contained two units of acid phosphatase (0.26 mU/µl, Sigma Aldrich) and were incubated for 30 min at 37°C, to generate deoxynucleotides. 30 µl of the sample was injected into an Acquity UPLC (Waters, USA) HPLC system running thermo C18 column 100×2.1 mm, followed by particle 5 µm quadrupole tandem mass spectrometer (Applied Biosystems TRIPLE QUAD 5500). Analyst 1.5.2.A step gradient program was applied to separate all the analytes with a flow rate of 300 ml/min. The mobile phase consisted of methanol (component A) and 20 mmol/l ammonia acetate buffer at pH 4.5 (component B). After separation, the analytes in the HPLC efferent were introduced into the mass spectrometer through a TurboIonspray interface coupled with a heated turbo nitrogen stream to evaporate solvents and to increase ionization efficiency. The following mass transitions were monitored–dA: 252.2/234.1; dA1∶252.2/96.0; dG: 268.1/232.1; dG1∶268.1/184.1; dG2∶268.1/124.1. A and A1 are derivatives of dATP and G, G1 and G2 are derivatives of dGTP.

## Results

### Identification of Itu as a p53 Activator

**Figure 1:**
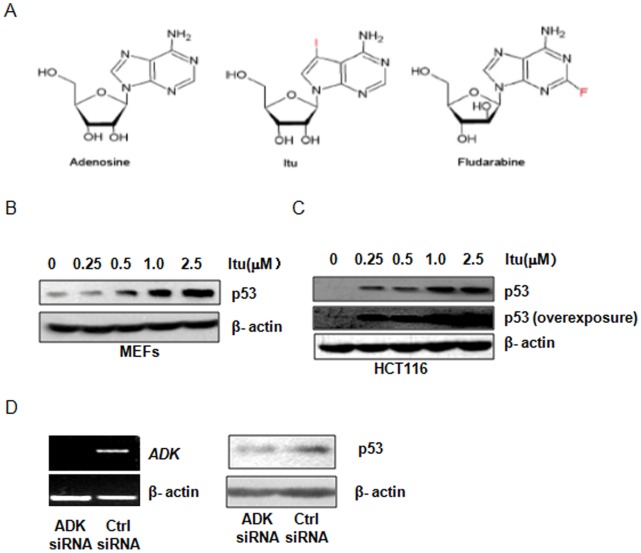
Identification of Itu as a p53 activator. A. The structures of Itu, adenosine, and Fludarabine. B. Western blot shows that Itu up-regulates p53 in MEFs in a dose-dependent manner. Cells were treated with various concentrations of Itu for 8 hrs and the levels of p53 were analyzed by western blot. C. Western blot shows that Itu up-regulates p53 in HCT116 cells in a dose-dependent manner. Cells were treated with various concentrations of Itu for 8 hrs and the levels of p53 were analyzed by western blot. A longer-exposed film was also shown to indicate that p53 was expressed in HCT116 cells at the basal level. D. Knock-down of ADK did not activate p53. Left panel: the decrease of ADK mRNA in the presence of ADK siRNA; Right panel: the protein levels of p53 in the presence of control and ADK siRNA in HCT116 cells.

To search for p53 activators, we screened by western blot analysis a panel of kinase inhibitors and a panel of phosphatase inhibitors to look for known compounds that could up-regulate p53 at the protein levels (for the list of the inhibitors, see Table S1) [17]. Primary MEFs were used for the screening because cell lines usually carry mutations in p53 and/or its upstream regulators. These inhibitors were applied at 10 µM, the concentration recommended by the manufacturer for inhibition of kinases or phosphatases. Two compounds out of the 113 inhibitors were able to induce p53 expression at the protein levels. One is 5-Iodotubercidin (Fig. 1A), a compound that is used as an inhibitor for adenosine kinase and other kinases such as Erk2. Itu has been shown to have anticonvulsant activity as well [19]. Itu-induced p53 up-regulation was observed in both MEFs and HCT116, with the latter being a colon cancer cell line positive for p53 (Fig. 1B and 1C). Dosage experiments indicated that Itu was able to up-regulate p53 at concentrations as low as 0.25 µM (Fig. 1B and 1C).

How does Itu induce p53 up-regulation? p53 expression is up-regulated by various stress and oncogene activation, especially genotoxic stress [25]. Since Itu is a widely used inhibitor of ADK, which plays a critical role in adenosine homeostasis [26], [27], it is possible that Itu disrupts adenosine homeostasis, interferes with DNA synthesis and causes DNA damage and thus activates p53. If this is true, knocking down ADK would mimic ADK inhibition with Itu in up-regulating p53. We used pooled siRNA to knock down ADK in HCT116 cells (Fig. 1D, left panel), and found that the decrease of ADK levels, unlike inhibition of ADK with Itu, did not significantly alter the protein levels of p53 at the basal level (Fig. 1D, right panel), suggesting that Itu-induced p53 up-regulation is not likely caused by direct inhibition of ADK.

### Itu Causes DNA Damage

**Figure 2:**
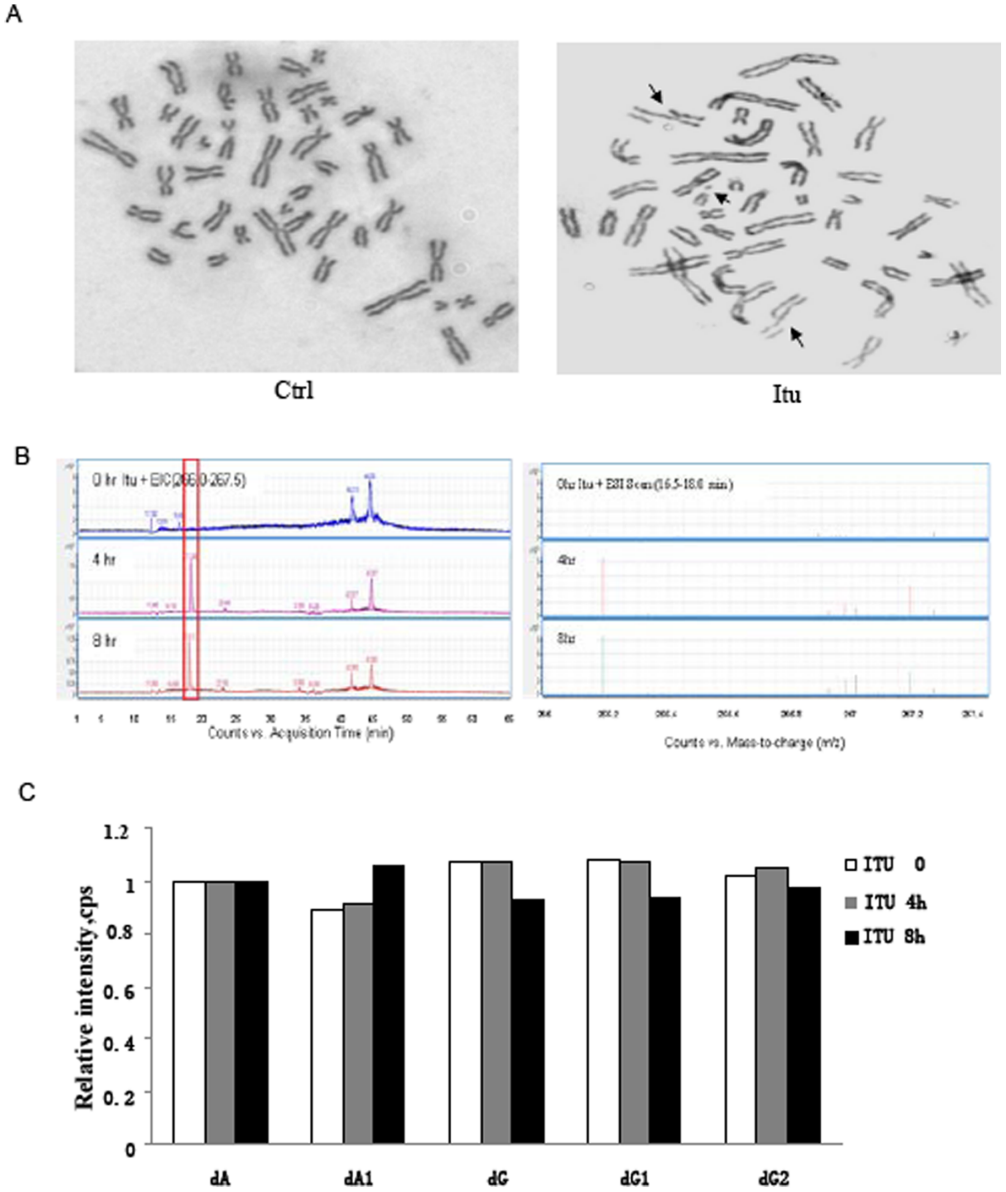
Itu generates DNA damage. A. Representative pictures of the karyotypes of HCT116 in the absence or presence of Itu. B. Detection of Itu derivative in genomic DNA in replicating cells. HPLC-MS analysis of Itu metabolites in the genomic DNA of Itu-treated cells. Genomic DNA was isolated from Itu-treated cells and digested into nucleosides, which were analyzed with HPLC (left panel), which was directly linked to mass spectrometer. MS analysis of the molecule with the size of dTU was shown at the right panel. C. The intracellular dATP and dGTP pools were not affected by Itu treatment. A and A1 are derivatives of dATP and G, G1 and G2 are derivatives of dGTP.

Itu has a purine-like structure with “N-H” being replaced by “C-I” at the 7^th^ position of the purine nucleoside (Fig. 1A). Itu might be incorporated into DNA after being converted to deoxyribose by ribonucleotide reductase [4]. Itu theoretically could form 2 hydrogen bonds with thymidine in double stranded DNA (Fig. S1). However, Itu-T pairing can cause a spacing problem as “C-I” of Itu (position 7) is much bigger than “N-H” of adenosine, and the altered purine ring may not be recognizable by DNA polymerases. Moreover, “C-I” of Itu is rather active and unstable. Itu is thus likely to be metabolized and subsequently incorporated into DNA to cause DNA damage. To confirm this, we first tested whether Itu could alter the karyotypes of HCT116 cells. The cells were first treated with Itu for 36 hrs and then with spindle poison colchicine to condense the chromosomes. The number and appearance of the chromosomes were analyzed under a light microscope after gimsa staining. It is obvious that Itu treatment led to the appearance of broken chromosomes in 32% of Itu-treated cells compared to 0% in untreated cells (Fig. 2A), suggesting that Itu could cause double stranded DNA breaks.

Some of the chemotherapeutic drugs work by intercalating DNA (e.g., doxorubicin) or crosslink DNA (e.g., cisplatin), while nucleoside analogs generate DNA breaks by being incorporated into DNA and disrupting nucleotide pairing. To test whether Itu might be metabolized and incorporated into genomic DNA during replication, we directly analyzed the nucleotides derived from genomic DNA isolated from Itu-treated cells that were either in the stationary phase or the log phase, with HPLC-mass spectrometer. The identity of the nucleosides was confirmed by comparing to corresponding reference substance. Itu (MW 392.153) can be metabolized into deoxy-iodotubercidin (dITU, MW 376.154), tubercidin (TU (with the unstable -I removed), MW 266.257), and deoxy-tubercidin (dTU, MW 250.257). We first compared the nucleotides derived from the genomic DNA of HCT116 and MEFs and found that HCT116 showed a much more complicated spectrum of nucleotides and their derivatives than those of MEFs, suggesting that nucleotides metabolism might be altered in cancer cells. We then decided to use MEFs to test whether Itu derivatives are incorporated into DNA. We found that dTU was present in the genomic DNA of Itu-treated log phase cells but not in stationary cells or untreated cells (Fig. 2B). These results suggest that Itu is metabolized (with -I removed) and incorporated into the DNA.

Several studies have shown that purine analogs such as fludarabine and clofarabine could inhibit ribonucleotide reductase, an enzyme that catalyzes the conversion of ribonucleotides to deoxyribonucleotides, thus decreasing the levels of cellular dNTPs and interfering with DNA synthesis [4]. We found that treatment with Itu at doses sufficient to upregulate p53 showed no effect on the mRNA levels of ribonucleotide reductase. We also compared the pools of dATP and dGTP, which are products of ribonucleotide reductase, in control and Itu-treated cells with mass spectrometry following an established protocol, and found that Itu treatment did not affect the pools of dATP or dGTP in the cells (Fig. 2C). These findings suggest that Itu does not inhibit ribonucleotide reductase.

**Figure 3:**
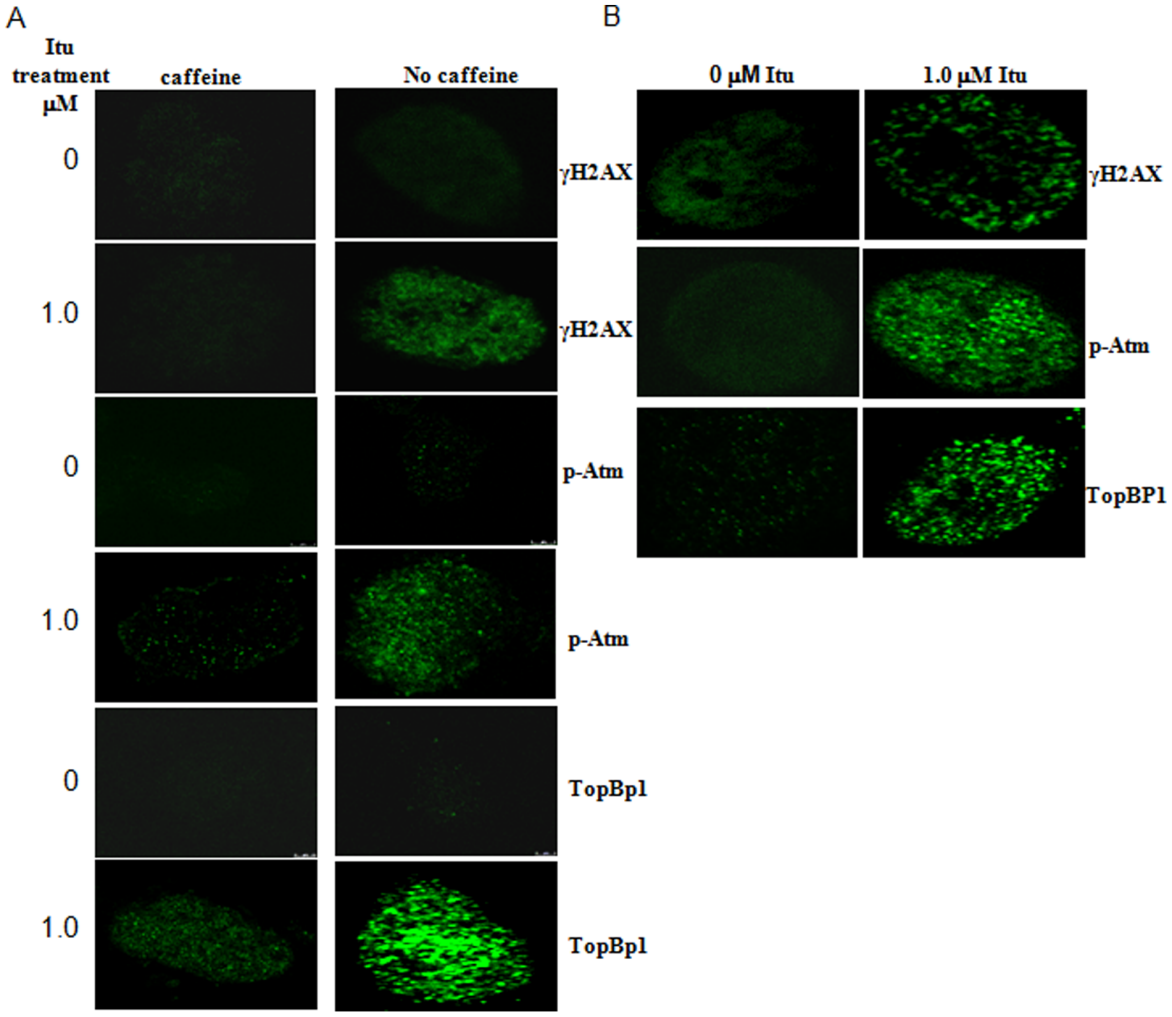
Itu generates DNA damage-induced nuclear foci. A. Treatment of HCT116 with 1 µM of Itu for 8 hrs induced nuclear foci positive for γH2AX, TopBP1, and activated Atm. Caffeine treatment diminished the formation of these foci. HCT116 cells were pretreated with caffeine (5 mM) for 1 hr and then Itu was added to the cultures for 8 more hrs. Cells were the fixed and stained for γH2AX, TopBP1, or p-Atm. B. Treatment of MEFs with 1 µM of Itu for 8 hrs induced nuclear foci positive for γH2AX, TopBP1, and activated Atm.

To validate this finding, we treated HCT116 and MEFs with Itu for 8 hrs and analyzed the formation of DNA damage-induced nuclear foci, which are postulated as DNA damage and repair centers [28], [29]. Among the earliest proteins assembled at the DNA breaks are γH2AX, a histone H2A variant that is ubiquitously expressed. It can be phosphorylated by PIKK members such as Atm and Atr quickly after DNA damage [30]. We did find that Itu treatment resulted in an accumulation of nuclear foci positive for γH2AX in both HCT116 (10.39±1.82% for untreated cells vs. 90.19±2.21% for Itu treated cells) and MEFs (7.45±0.29% for untreated cells vs. 91.63±0.73% for Itu treated cells) (Fig. 3A and 3B). Moreover, Itu treatment also resulted in an accumulation of nuclear foci positive for TopBP1, another foci-localized protein, in HCT116 (14.25±5.12% for untreated cells vs. 95.24±1.39% for Itu treated cells) and MEFs (13.08±5.15% for untreated cells vs. 93.32±2.70% for Itu treated cells) (Fig. 3A and 3B).

### Itu Activates Atm

**Figure 4:**
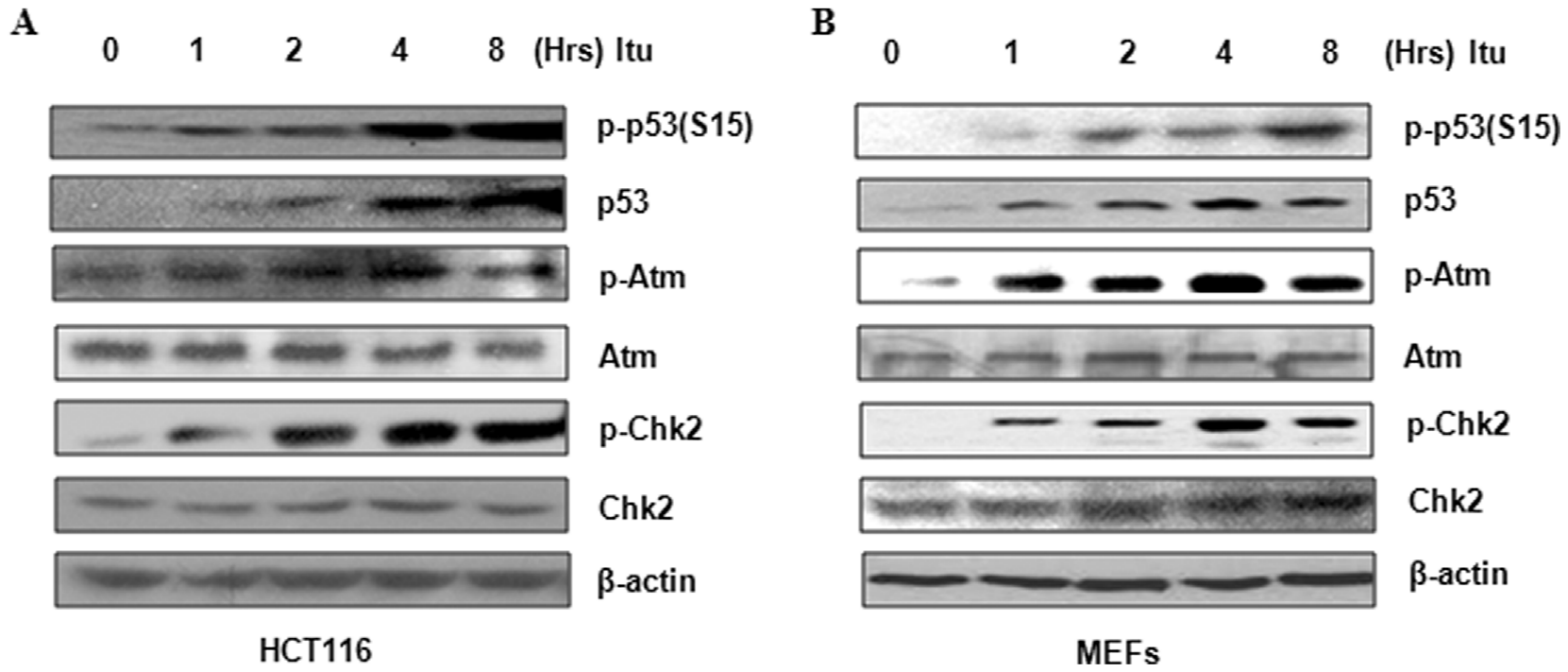
Itu treatment activates Atm and Chk2 and leads to p53 S15 phosphorylation. A. HCT116 cells were treated with 1 µM Itu for different periods of time and activation of Atm, Chk2, and p53 was assessed with western blot using specific phospho-antibodies. B. MEFs cells were treated with 1 µM Itu for different periods of time and activation of Atm, Chk2, and p53 was assessed with western blot using specific phospho-antibodies.

In addition to foci positive for γH2AX and TopBP1, Itu treatment also resulted in nuclear foci positive for p-Atm (10.13±2.88% for untreated cells vs. 85.84±4.63% for Itu treated cells) (S1981 phosphorylation, an indication of Atm activation) (Fig. 3A and 3B) [31], indicating that Itu causes DNA damage and activates Atm. Consistent with this observation, Itu-induced p-Atm and γH2AX foci formation in HCT116 cells could be diminished (down to 15.37±1.15% and 9.00±1.19% respectively) by treatment with caffeine, an inhibitor of Atm and Atr (Fig. 3A). To substantiate the finding that Itu activates Atm, we used western blot to analyze Atm S1981 phosphorylation, as well as Atm-mediated Chk2 phosphorylation. Although Atm is thought to be only activated by double stranded DNA breaks [7], [31], [32], recent studies showed that nucleoside analogs can also activate Atm as well as its downstream pathways [30], [33], [34]. Atm is found to be localized at the stalled replication forks and may help to prevent fork collapse in the cells [30], [33]. We found that Itu treatment led to a time-dependent phosphorylation on S1981 of Atm as well as Chk2 phosphorylation at Thr68 in HCT116 cells (Fig. 4A). Itu treatment also led to Ser15 phosphorylation on p53 (Fig. 4A), which is carried out by PIKKs as well. Similarly, Itu could stimulate p53 phosphorylation and Chk2 phosphorylation in MEFs (Fig. 4B). These results further support the conclusion that Itu causes DNA damage and activates Atm.

### Itu Led to S Phase Extension and G2/M Arrest in a p53-dependent Manner

**Figure 5:**
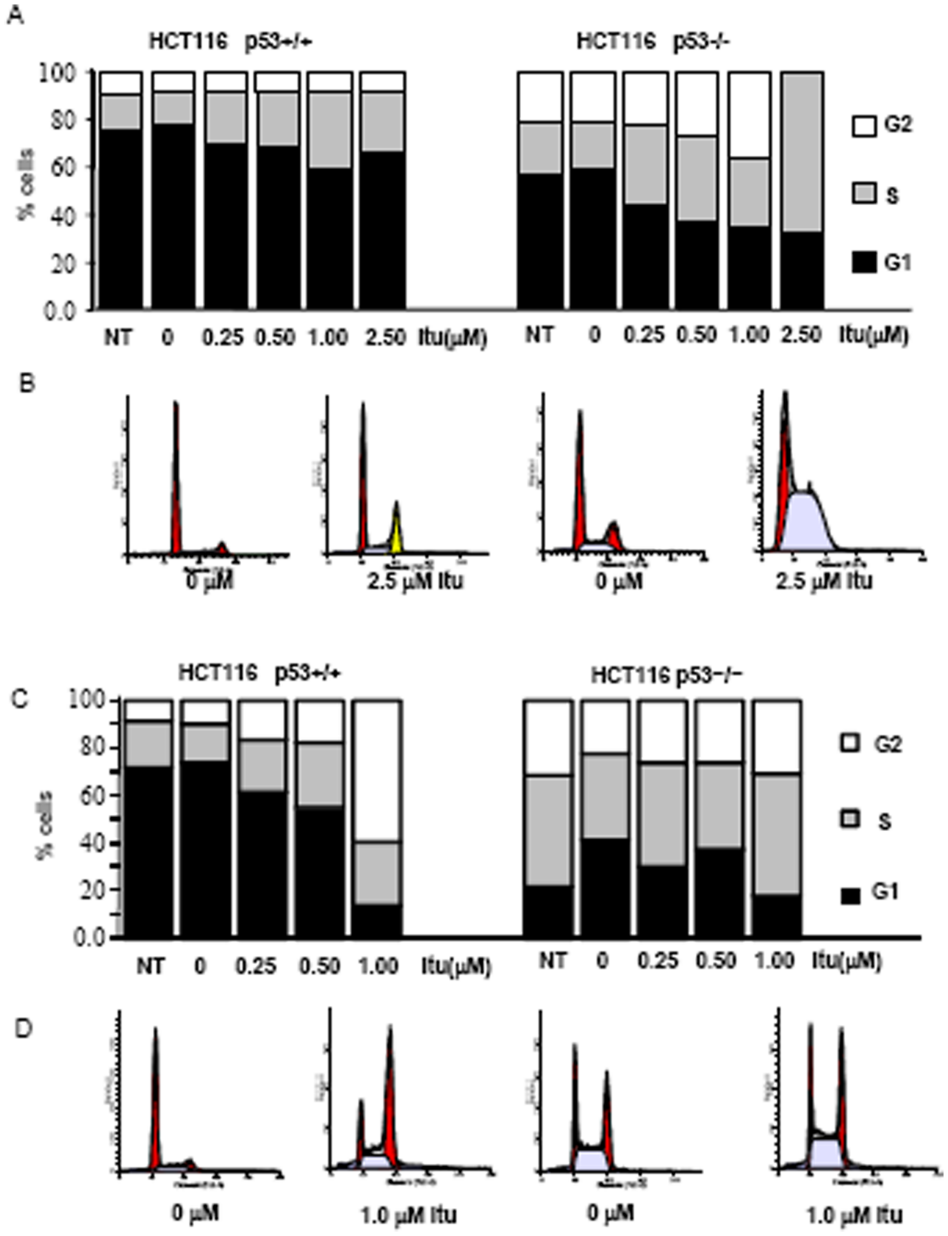
Itu treatment results in an increase in S and G2/M phase cells. A. HCT116 (p53+/+ and p53−/−) were treated with different concentrations of Itu for 24 hrs. The cells were fixed, stained with PI, and analyzed with FACS. Percentage of cells in different phases was shown. B. Representative cell cycle profiles of p53+/+ and p53−/− HCT116 in the presence of 2.5 µM of Itu for 24 hrs. C. HCT116 (p53+/+ and p53−/−) were treated with different concentrations of Itu for 48 hrs. The cells were fixed, stained with PI, and analyzed with FACS. Percentage of cells in different phases was shown. D. Representative cell cycle profiles of p53+/+ and p53−/− HCT116 in the presence of 1.0 µM of Itu for 48 hrs.

Many nucleoside analogs cause cell cycle arrest at the S phase with exceptions such as 20-C-cyano-20-deoxy-1-β-D-arabino-pentofuranosylcytosine (CNDAC), which causes cell cycle arrest at the G2 phase [4], [35]. Incorporation of nucleoside analogs terminates the elongation of nascent DNA strand and leads to stalling of replication forks. To test whether Itu activates cell cycle checkpoints, we treated p53+/+ and p53−/− HCT116 cells with different concentrations of Itu for 24 or 48 hrs. It was found that 24 hr-treatment with higher concentrations of Itu led to a modest increase in the percentage of S phase cells, which was accompanied with a decrease in the percentage of G1 phase cells, without significantly altering the percentage of G2/M phase cells (Fig. 5A and 5B). When the cells were treated with various concentrations of Itu for 48 hrs, more cells were in the G2 phase, accompanied by a decrease in G1 phase but no significant change in the percentage of S phase cells (Fig. 5C and 5D). One explanation is that cells were initially stuck in the S phase but managed to pass through S phase and were eventually blocked in the G2/M phase. Thus Itu mainly activates the G2 checkpoint. This is in contrast to most of the nucleoside analogs, which lead to S phase cell cycle arrest [4], and ionizing radiation (IR), which induces cell cycle arrest at G1 and G2 phase and is accompanied by a decrease in S phase in HCT116 cells [21]. In the absence of p53, 24-hr-treatment led to an initial increase in the S phase at higher doses of Itu (Fig. 5A and 5B), yet 48 hrs after treatment, the difference between control and treated cells became minimal (Fig. 5C and 5D), indicating that p53−/− HCT116 cells, but not the p53+/+ HCT116 cells, undergo S phase arrest and that p53−/− cells managed to escape this checkpoint. Moreover, p53−/− cells did not show a G2 phase arrest (Fig. 5A and 5B). These results indicate that Itu-induced G2 phase arrest requires the presence of p53.

### Itu Induces p53-dependent and -independent Cell Death

**Figure 6:**
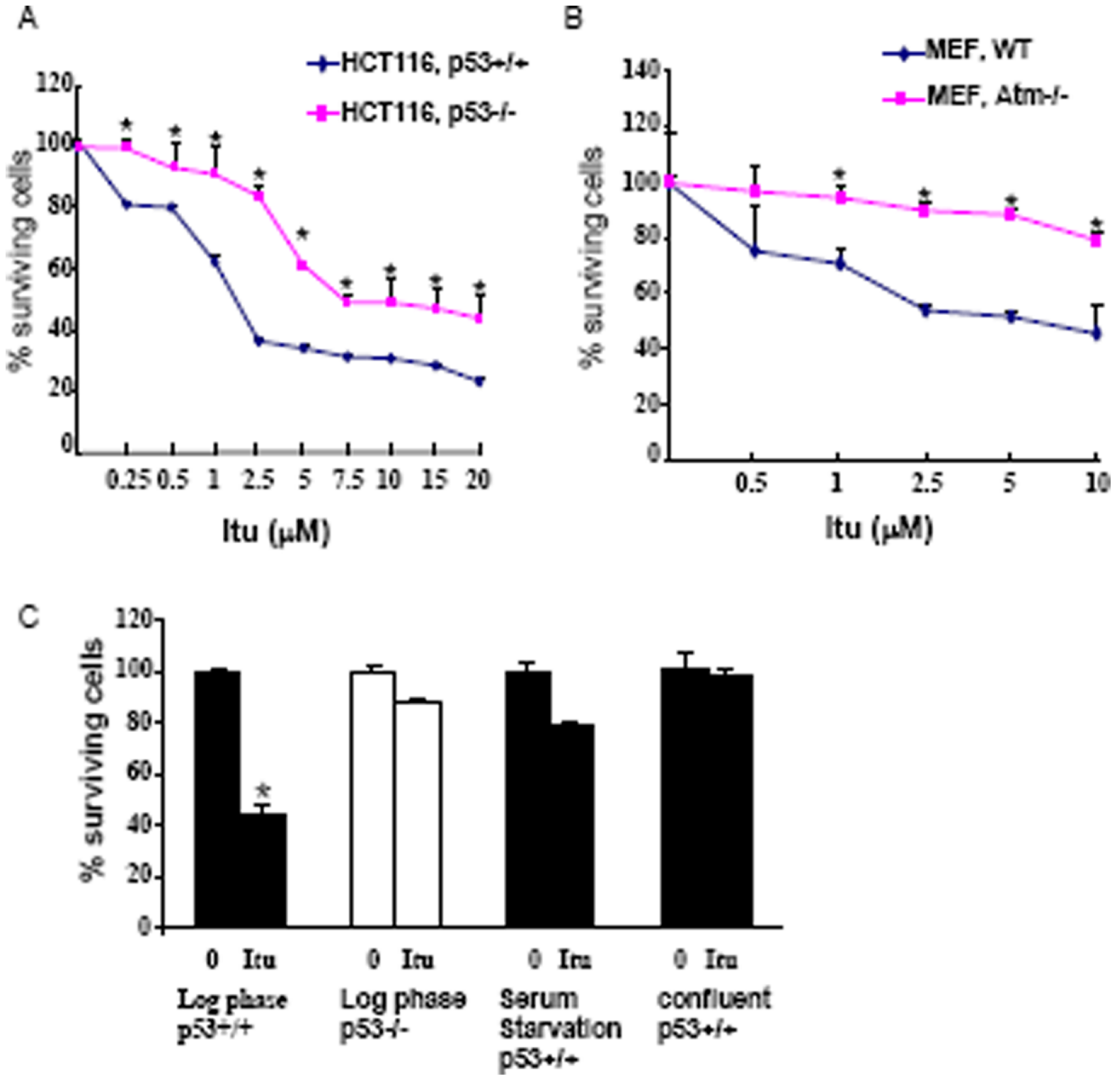
Itu induced cell death in p53-dependent and independent manners. A. HCT116 cells (p53+/+ and p53−/−) were treated with various concentrations of Itu for 48 hours and the number of cells were measured by Wst-1 assay. *p<0.05 when p53−/− cells were compared to p53+/+ cells. B. Atm+/+ and Atm−/− MEFs were treated with various concentrations of Itu for 48 hours and the number of cells were measured by Wst-1 assay. *p<0.05 when Atm−/− cells were compared to Atm+/+ cells. C. Wild type and p53−/− HCT116 cells were treated with Itu for 24 hours and the number of cells was measured by Wst-1 assay. Itu did not induce cell death when cells were cultured in 0.1% serum or confluent in wild type HCT116 cells. *p<0.05 when compared to untreated cells.

p53 has a strong proapoptotic role and it has been well established that DNA damage induces cell death mainly through the Atm-p53 pathway. We then tested the cytotoxicity of Itu in HCT116 and the involvement of Itu-induced p53 up-regulation. Itu treatment led to cell death in p53+/+ HCT116 cells (EC50 = 1.88 µM), yet p53−/− HCT116 cells showed resistance to Itu-induced cell death (EC50 = 7.8 µM) (Fig. 6A), suggesting an important role for p53 in mediating Itu-induced cell death. Moreover, Atm deficiency, which is known to diminish p53 induction, also inhibited Itu-induced cell death in MEFs (Fig. 6B) [36], consistent with the well-accepted role for Atm in promoting cell death under genotoxic stress. The less-than complete rescue of cell death by p53 deficiency suggest that Itu might possess cytotoxicity that is independent of p53, for example, by inhibiting pro-survival signaling such as Erk2.

Many of the nucleoside analogs require DNA incorporation to execute its cytotoxic effect. To further test the cytotoxic effect of Itu on non-dividing cells, we cultured HCT116 either to stationary phase or in the presence of 0.1% serum for 24 hrs, with most of the cells being in the G1 phase. Under these conditions, Itu failed to kill the cells (Fig. 6C), suggesting that Itu needs to be incorporated into DNA in order to execute its cytotoxic activity. This is consistent with our observation that Itu metabolite is present in the genomic DNA of replicating cells (Fig. 2B).

However, flow cytometry assays did not reveal a significant increase in the sub-G1 phase cells, an indication of apoptotic cells, after 24 or 48 hrs of Itu-treatment with various doses (Fig. 5B and 5D). This is in contrast to IR or HADC (histone deacetylases) inhibitor MGCD0103, which led to a marked increase in the sub-G1 phase in HCT116 cells [21], [37]. Moreover, no DNA ladders were observed after Itu treatment (data not shown). These results suggest that Itu does not induce the classical apoptosis pathway in HCT116, although Itu-induced cell death still largely depends on p53. Previously studies have shown that some of the nucleoside analogs kill cells not via the classical apoptotic pathway [4]. These results suggest that Itu may affect cell death/survival via multiple pathways, including p53-dependent and -independent pathways.

### Itu has Anti-tumor Activity

**Figure 7:**
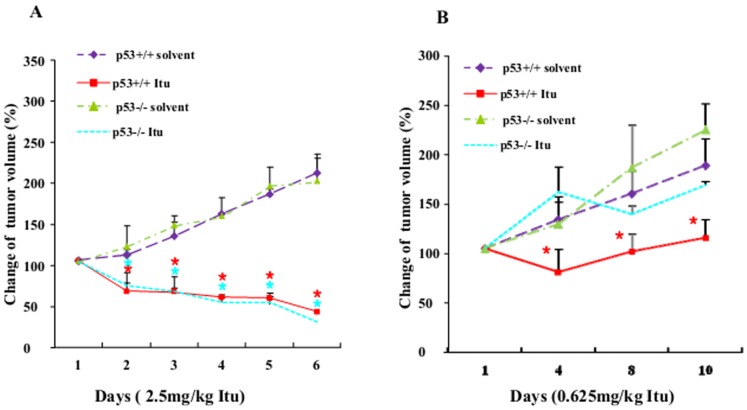
Itu showed anti-tumor activity. A. High dose of Itu resulted in tumor shrinkage in nude mice. p53+/+ and p53−/− HCT116 was injected into nude mice. Two weeks later, Itu was ip injected every day and the tumor size was measured. (n = 5) The tumor volumes at various days were normalized to that of day 1, which was set at 100%. *, p<0.05 when comparing Itu treatment with solvent injection. B. Low dose of Itu resulted in tumor shrinkage in nude mice. p53+/+ and p53−/− HCT116 was injected into nude mice. Two weeks later, Itu was ip injected every day and the tumor size was measured. (n = 5) The tumor volumes at various days were normalized to that of day 1, which was set at 100%. *, p<0.05 when comparing Itu treatment with solvent injection.

The above experiments show that Itu is a genotoxic drug that can inhibit cell proliferation and induce cell death in p53-dependent and independent manners. As such, Itu is likely to have anti-tumor activity. To test this possibility, we used HCT116-initiated colon carcinoma xenograft in nude mice. We found that Itu at 2.5 mg/kg induced rapid tumor regression (Fig. 7A), while the control group still showed marked tumor growth. At this dose, Itu treatment also led to a decrease in the body weights of the mice (down by 6% at the end of treatment), suggesting that Itu has an adverse side effect in vivo. Surprisingly, at this dose of Itu, p53 seems not to play an important role, as p53−/− HCT116-initiated tumor was repressed as well by Itu (Fig. 7A). We then lowered the dose of Itu to 0.625 mg/kg and repeated the experiments. Itu stilled showed an inhibitory effect on tumor growth, yet p53−/− tumors showed resistance to Itu treatment (Fig. 7B). At this dose, the body weights of these mice were not significantly affected. Thus, at low doses, Itu inhibits tumor growth via p53; however, at higher doses, p53-independent pathways might play an important role.

## Discussion

Itu has been used as a general inhibitor of various kinases [18], including PKA (IC50 = 5–10 µM), phosphorylase kinase (IC50 = 5–10 µM), casein kinase I (IC50 = 0.4 µM) and II (IC50 = 10.9 µM), PKC (IC50 = 0.4 µM), ERK2 (IC50 = 0.525 µM), and adenosine kinase (IC50 = 26 nM). The IC50 for most of the kinases inhibited by Itu is higher than 0.25 µM, which is shown to be sufficient to up-regulate p53. Itu also initiates glycogen synthesis by activating glycogen synthase and inactivating phosphorylase. As such, Itu increases fatty acid oxidation activity of the liver at the expense of lipogenesis [38], [39]. Due to its critical role in inhibiting ADK and controlling the intracellular and extracellular adenosine levels, Itu has been tested as a potential treatment for disorders of the immune, cardiovascular, and nervous systems in animal models. ADK inhibitors could increase the intravascular adenosine concentrations and thus act as anti-inflammatory agents [40]. Pretreatment with Itu (1 mg/kg) has been reported to limit the development of heart infarct [41], [42]. Since adenosine is an inhibitory modulator of brain activity with neuroprotective and anticonvulsant properties, dysfunction of the adenosine system is found to be involved in pathologies ranging from epilepsy to neurodegenerative disorders and psychiatric conditions [27], [43]. Transient down-regulation of ADK after acute brain injury protects the brain from seizures and cell death, while chronic ADK overexpression causes seizures in epilepsy and promotes cell death in epilepsy and stroke [44]–[46]. Based on these findings, it has been suggested that ADK is a therapeutic target for epilepsy and stroke and that Itu might be a potential drug to treat these disorders.

This study, for the first time, identified Itu as a genotoxic drug and a potential anti-tumor agent, in addition to its role as a kinase inhibitor. We found that i) Itu metabolite could be incorporated into DNA and cause DNA lesions, evidenced by formation of γH2AX and TopBP1 positive nuclear foci and chromosomal breakage; ii) Itu activates Atm, evidenced by S1981 phosphorylation on Atm detected by nuclear foci formation and western blot, and phosphorylation of Chk2; iii) Itu activates p53, evidenced by p53 up-regulation and S15 phosphorylation; iv) Itu causes p53-dependent cell cycle arrest and cell death; v) Itu inhibits tumor growth in carcinoma xenograft mouse models in a p53-dependent manner at low doses. In addition, our cell death and xenograft studies indicate that Itu also executes anti-growth and anticancer activities via p53-independent mechanisms, which may include p53 redundant pathways as well as inhibition of various kinases. It is also likely that the Atm-p53 pathway crosstalks with the p53-independent pathways to decide the cell fates in response to Itu treatment. Further studies will be needed to identify these p53-independent pathways activated by Itu. Nonetheless, our study convincingly demonstrates that Itu is genotoxic drug that activates the classical Atm-p53 pathway.

Due to its ability to disrupt genome integrity and the findings that genotoxic drugs can cause cancer or aging in various organisms [47], [48], the uses of Itu for treatment of any diseases might be risky, unless used at very low levels. Moreover, caution needs to be exercised when using Itu as an inhibitor for adenosine kinase and Erk2, as multiple pathways can be activated by Itu-induced DNA damage. On the other hand, our findings suggest that Itu might be a potential chemotherapeutic drug, although more studies will be needed to compare the anti-tumor efficacy of Itu with other purine/pyrimidine derivatives. Moreover, the ability of Itu to cause G2 phase arrest might provide a basis for combinational use of drugs with S phase and G2 phase blocking abilities.

How does Itu cause DNA damage? Nucleoside analogs that are used in treatment of solid tumors and hematological malignancies are usually incorporated into DNA and thus block the extension of nascent DNA strand and eventually cause stalling of replication. Some of these drugs can also dysregulate the deoxynucleotide pool balance and thus affect DNA synthesis [4]. We detected Itu metabolite in the DNA of cells undergo DNA replication. This, together with our findings that Itu failed to kill non-dividing cells and that Itu could induce the formation of γH2AX positive foci and chromosomal breakage, support the theory that Itu causes DNA damage by being incorporated into the DNA after metabolized, where it may disrupt base pairing. On the other hand, we excluded ADK, a major target of Itu and a regulator of adenosine levels, as a mediator of Itu-induced p53 up-regulation, as we found that knock-down of ADK did not up-regulate the expression of p53. Moreover, we also excluded a possible role for ribonucleotide reductase in Itu-mediated DNA damage.

In summary, this study identified general kinase inhibitor Itu as a genotoxic drug that activates the Atm-p53 pathway and has anti-tumor activity. Itu is unique compared to other nucleoside analogs in the way that it induces G2 arrest in a p53 -dependent manner. Moreover, at higher doses, Itu might activate p53-independent pathways, which may cooperate with p53 to kill cells and inhibit tumor growth. Our data support the concept that incorporation of Itu metabolite into DNA causes DNA breaks, which triggers the DNA damage response. Overall, these findings suggest that Itu might be a potential chemotherapeutic drug with distinct working mechanisms.

## Supporting Information

Figure S1 **Proposed base-pairing between Itu and T.**
(TIF)

Table S1 **Phophatase and Kinase inhibitors.**
(DOC)
