# Supplementary figures and images for: Identification of 5-Iodotubercidin as a Genotoxic Drug with Anti-Cancer Potential
Source: PLoS One. 2013 May 7;8(5):e62527. doi: 10.1371/journal.pone.0062527 (PMC3646850; doi:10.1371/journal.pone.0062527)

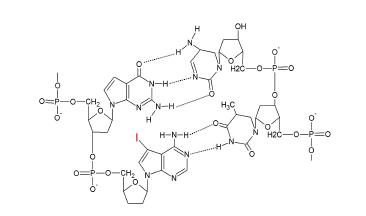

Supplement: Figure S1 — Proposed base-pairing between Itu and T. (TIF) [file pone.0062527.s001.tif]
