# Supplementary material for: Identification of 5-Iodotubercidin as a Genotoxic Drug with Anti-Cancer Potential
Source: PLoS One. 2013 May 7;8(5):e62527. doi: 10.1371/journal.pone.0062527 (PMC3646850; doi:10.1371/journal.pone.0062527)
Supplement: Table S1 — Phophatase and Kinase inhibitors. (DOC) [file pone.0062527.s002.doc]

**Supporting Information**

**Table S1**. **Phophatase and Kinase inhibitors**

| **PHOSPHATASE INHIBITORS** | **TARGET** |
| --- | --- |
| Cantharidic acid | PP1 and PP2A |
| Cantharidin | PP1 and PP2A |
| Endothall | PP2A |
| Benzylphosphonic acid | Tyrosine phosphatases |
| L-p-Bromotetramisole oxalate | Tyrosine phosphatases |
| RK-682 | Tyrosine phosphatases |
| RWJ-60475 | CD45 tyrosine phosphatase |
| RWJ-60475(AM)3 | CD45 tyrosine phosphatase(cell permeable) |
| Levamisole HCl | Mammalian alkaline phosphatase |
| Tetramisole HCl | Mammalian alkaline phosphatase |
| Cypermethrin | Calcineurin(PP2B) |
| Deltamethrin | Calcineurin(PP2B) |
| Fenvalerate | Calcineurin(PP2B) |
| Tyrphostin 8 | Calcineurin(PP2B) |
| CinnGEL | PTP1B |
| CinnGEL 2 Me | PTP1B(cell permeable) |
| BN-82002 | CDC25 |
| Shikonin | TPEN |
| NSC-663284 | CDC25 |
| Cyclosporin A | Calcineurin(PP2B) |
| pentamidine | PRL1 |
| BVT-948 | Tyrosine phosphatases |
| B4-Rhodanine | PRL3 |
| BML-268 | PTP1B |
| Dioxophenanthrene | CD45 tyrosine phosphatase |
| BML-260 | JSP-1 |
| PD-144795 | Calcineurin(PP2B) |
| BML-267 | PTP1B |
| BML-267 Ester | PTP1B(cell permeable) |
| OBA | Tyrosine phosphatases |
| OBA Ester | Tyrosine phosphatases(cell permeable) |
| Gossypol | Calcineurin(PP2B) |
| Alendronate | Tyrosine phosphatases |

| **KINASE INHIBITORS** | **TARGET** |
| --- | --- |
| PD-98059 | MEK |
| U-0126 | MEK |
| SB-203580 | p38 MAPK |
| H-7 | PKA,PKG,MLCK,and PKC |
| H-9 | PKA,PKG,MLCK,and PKC |
| Staurosporine | Pan-specific |
| AG-494 | EGFRK,PDGFRK |
| AG-825 | HER1-2 |
| Lavendustin A | EGFRK |
| RG-14620 | EGFRK |
| Tyrphostin 23 | EGFRK |
| Tyrphostin 25 | EGFRK |
| Tyrphostin 46 | EGFRK,PDGFRK |
| Tyrphostin 47 | EGFRK |
| Tyrphostin 51 | EGFRK |
| Tyrphostin 1 | Negative control for tyrosine  kinase inhibitors |
| Tyrphostin AG 1288 | Tyrosine kinases |
| Tyrphostin AG 1478 | EGFRK |
| Tyrphostin AG 1295 | Tyrosine Kinases |
| Tyrphostin 9 | PDGFRK |
| HNMPA(Hydroxy-2  -naphthalenylmethylphosphonic acid) | IRK |
| Damnacanthal | p56 lck |
| Piceatannol | Syk |
| PP1 | Src family |
| AG-490 | JAK-2 |
| AG-126 | IRAK |
| AG-370 | PDGFRK |
| AG-879 | NGFRK |
| LY 294002 | PI3-K |
| Wortmannin | PI3-K |
| GF 109203X | PKC |
| Hypericin | PKC |
| RO-31-8220 | PKC |
| Sphingosine | PKC |
| H-89 | PKA |
| H-8 | PKA,PKG |
| HA-1004  HA-1077 | PKA,PKG  PKA,PKG |
| HDBA(2-Hydroxy-5-(2,5-  dihydroxybenzylamino)benzoic acid) | EGFRK,CaMK II |
| KN-62 | CaMK II |
| KN-93 | CaMK II |
| ML-7 | MLCK |
| ML-9 | MLCK |
| 2-Aminopurine | p58 PITSLRE beta1 |
| N9-Isopropyl-olomoucine | CDK |
| Olomoucine | CDK |
| iso-Olomoucine | Negative control for olomoucine |
| Roscovitine | CDK |
| 5-Iodotubercidin | ERK2,adenosine kinase,CK1,CK2 |
| LFM-A13 | BTK |
| SB-202190 | p38 MAPK |
| PP2 | Src family |
| ZM336372 | cRAF |
| SU 4312 | Flk1 |
| AG-1296 | PDGFRK |
| GW 5074 | cRAF |
| Palmitoyl-DL-carnitine Cl | PKC |
| Rottlerin | PKC delta |
| Genistein | Tyrosine Kinases |
| Daidzein | Negative control for Genistein |
| Erbstatin analog | EGFRK |
| Quercetin dihydrate | PI3-K |
| SU 1498 | Flk1 |
| ZM 449829 | JAK-3 |
| BAY 11-7082 | IKK pathway |
| DRB(5,6-Dichloro-1-β-D  -ribofuranosylbenzimidazole) | CK II |
| HBDDE(2,2',3,3',4,4'-Hexahydroxy  -1,1'-biphenyl-6,6'-dimethanol dimethyl ether) | PKC alpha,PKC gamma |
| SP 600125 | JNK |
| Indirubin | GSK-3beta,CDK5 |
| Indirubin-3'-monoxime | GSK-3beta |
| Y-27632 | ROCK |
| Kenpaullone | GSK-3beta |
| Terreic acid | BTK |
| Triciribine | Akt signaling pathway |
| BML-257 | Akt |
| SC-514  BML-259 | IKK2  Cdk5/p25 |
| Apigenin | CK-II |
| BML-265(Erlotinib analog) | EGFRK |
| Rapamycin | mTOR |
